# Hypothermic Oxygenated Machine Perfusion Promotes Mitophagy Flux against Hypoxia-Ischemic Injury in Rat DCD Liver

**DOI:** 10.3390/ijms24065403

**Published:** 2023-03-11

**Authors:** Jia Luo, Yiqing Hu, Yinbiao Qiao, Haoyu Li, Jiacheng Huang, Kangdi Xu, Li Jiang, Hao Wu, Xiaoyi Hu, Junjun Jia, Lin Zhou, Haiyang Xie, Jianhui Li, Shusen Zheng

**Affiliations:** 1 Division of Hepatobiliary and Pancreatic Surgery, Department of Surgery, The First Affiliated Hospital, Zhejiang University School of Medicine, Hangzhou 310003, China; 2 NHC Key Laboratory of Combined Multi-Organ Transplantation, Hangzhou 310003, China; 3 Department of Hepatobiliary and Pancreatic Surgery, Shulan (Hangzhou) Hospital, Zhejiang Shuren University School of Medicine, Hangzhou 310015, China; 4 The Organ Repair and Regeneration Medicine Institute of Hangzhou, Hangzhou 310003, China; 5 Jinan Microecological Biomedicine Shandong Laboratory, Jinan 250117, China

**Keywords:** HOPE, DCD liver, mitophagy, hypoxia-ischemic injury, organ protection

## Abstract

Hypothermic oxygenated machine perfusion (HOPE) can enhance organ preservation and protect mitochondria from hypoxia-ischemic injury; however, an understanding of the underlying HOPE mechanism that protects mitochondria is somewhat lacking. We hypothesized that mitophagy may play an important role in HOPE mitochondria protection. Experimental rat liver grafts were exposed to 30 min of in situ warm ischemia. Then, grafts were procured, followed by cold storage for 3 or 4 h to mimic the conventional preservation and transportation time in donation after circulatory death (DCD) in clinical contexts. Next, the grafts underwent hypothermic machine perfusion (HMP) or HOPE for 1 h through portal vein only perfusion. The HOPE-treated group showed a better preservation capacity compared with cold storage and HMP, preventing hepatocyte damage, nuclear injury, and cell death. HOPE can increase mitophagy marker expression, promote mitophagy flux via the PINK1/Parkin pathway to maintain mitochondrial function, and reduce oxygen free radical generation, while the inhibition of autophagy by 3-methyladenine and chloroquine could reverse the protective effect. HOPE-treated DCD liver also demonstrated more changes in the expression of genes responsible for bile metabolism, mitochondrial dynamics, cell survival, and oxidative stress. Overall, HOPE attenuates hypoxia-ischemic injury in DCD liver by promoting mitophagy flux to maintain mitochondrial function and protect hepatocytes. Mitophagy could pave the way for a protective approach against hypoxia-ischemic injury in DCD liver.

## 1. Introduction

Organ shortage is a worldwide problem for transplantation development. The increasing application of marginal donor organs, such as DCD, accounts for a considerable proportion of transplantation, complementing the persisting organ shortage [1,2]. Despite DCD organs possibly resulting in inferior graft survival, increased rates of biliary ischemic cholangiopathy (IC) and primary nonfunction (PNF) are arising challenges for clinicians [3,4]. However, organ shortages have driven the development of novel methods for organ preservation and reconditioning [5,6], and new mechanical perfusion strategies are being pursued, including hypothermic [7], subnormothermic [8], and normothermic machine perfusion systems [9], which differ in their content and temperature [10,11]. Machine perfusion (MP) can improve the marginal graft quality, extend preservation time, and enable an assessment of their quality and viability, reducing needless organ waste [3].

HOPE is a dynamic preservation method under hypothermic conditions and the low-flow delivery of oxygen through the portal vein only or both the hepatic artery and portal vein (dual hypothermic oxygenated machine perfusion (D-HOPE)) [12]. This fairly straightforward and safe technique aims to safely preserve the liver, enhancing mitochondrial recovery; furthermore, it is also considered cheaper than normothermic machine perfusion (NMP) systems [3]. NMP is performed at a normal temperature (37 °C) to mimic the physiological environment and maintain organ metabolic activity [13]. Studies of transplantation showed a high protective effect of HOPE [14,15,16], which may enhance mitochondrial recovery and function [17]. HOPE was also promising in reducing the rates of biliary complications in DCD liver transplantations in clinical trials [4,16,18]. Although clinical applications of MP have been well demonstrated, precise mechanistic data are needed to maximize the benefits of MP technology.

Warm/cold ischemic and hypoxia injury in DCD grafts are contributing factors to post-transplantation syndrome, including oxidative stress, mitochondrial dysfunction, and adenosine triphosphate (ATP) depletion [19]. Mitochondrial reactive oxygen species (ROS) are the major messengers to spark autophagy [20,21]. Autophagy is a self-eating catabolic pathway conserved in eukaryotic cells, with the lysosome as the final destination, and it can be divided into three different types, namely, macroautophagy, microautophagy, and chaperone-mediated autophagy (CMA), depending on the different pathways and morphological characteristics of autophagy substrates [22]. According to the different uptake and degradation processes, autophagy can also be divided into selective and nonselective categories [23]. Autophagy may be impaired during the pathogenesis of ischemic reperfusion injury (IRI), resulting in poor tolerance to IRI [24]. Strategies that reduce ROS production and liver damage, and improve mitochondrial functionality and ATP secretion could help to expand the available pool for transplantation [25].

Mitochondria are commonly regarded as the batteries of the cell and are involved in signal regulation and producing ATP. Mitochondria form a network of highly dynamic organelles and undergo continual fission, fusion, and degradation [24]. Mitophagy is one of the organelle-specific selected macroautophagic pathways that refer to the removal of excess or damaged mitochondria to maintain mitochondrial homeostasis, and can be regarded as a process of degradation [26], with the damaged mitochondria leading to the initiation of selective mitophagy [27,28]. Thus, we hypothesized that mitophagy may play an important role in the protection of mitochondria by HOPE, and we sought to investigate the related mitophagy mechanism in a DCD rat model applied with HOPE to explore the possibility of mitophagy as a therapeutic target to improve graft quality in DCD transplantation.

## 2. Results

### 2.1. HOPE Alleviated DCD Liver Injury in Rats

**Figure 1:**
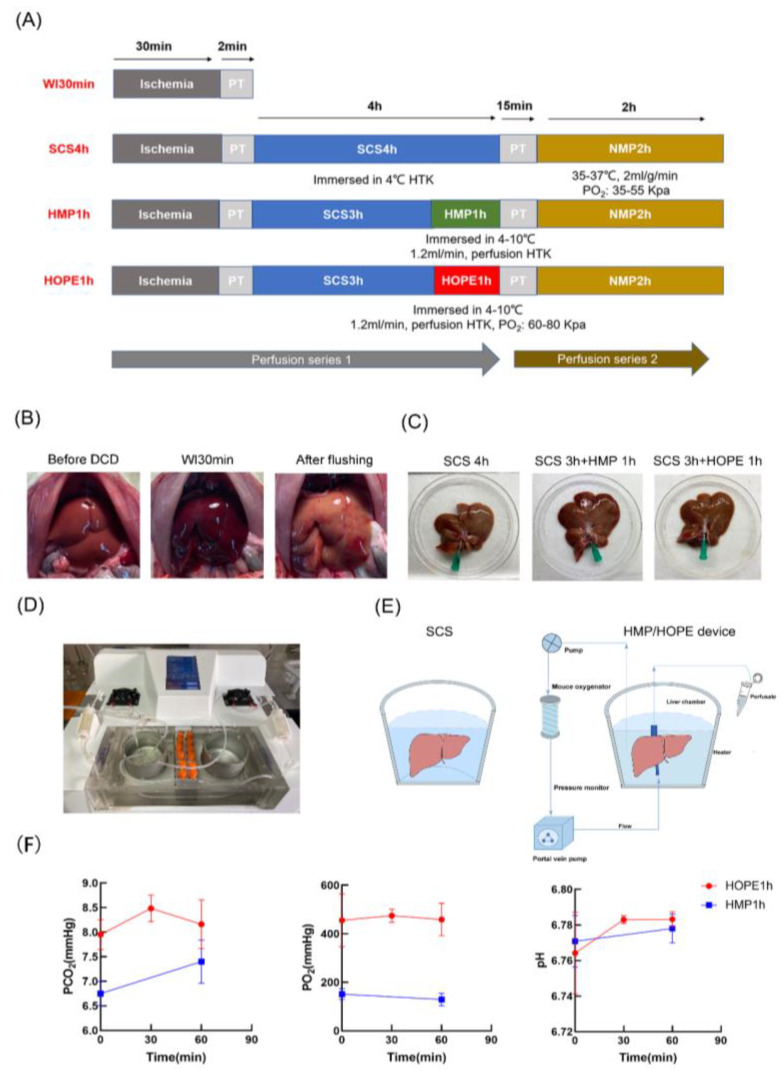
Four groups’ experimental protocols. (**A**) Perfusion series 1: liver subjected to 30 min warm ischemia in situ, flush, and static cold storage; and HMP or HOPE after static cold storage (*n* = 6 each). Perfusion series 2: livers except WI 30 min were perfused for 2 h using NMP to determine liver function and injury (*n* = 3 each). (**B**) Liver appearance before warm ischemia for 30 min and flush. (**C**) Liver morphology in three different hyperthymic storage or machine perfusion methods in perfusion series 1. (**D**) The machine system stored the livers and performed machine perfusion. (**E**) Schematic representation of SCS and MP devices. (**F**) The parameters of pH, PO_2_, and PCO_2_ of hyperthymic machine perfusion were calculated.

**Figure 2:**
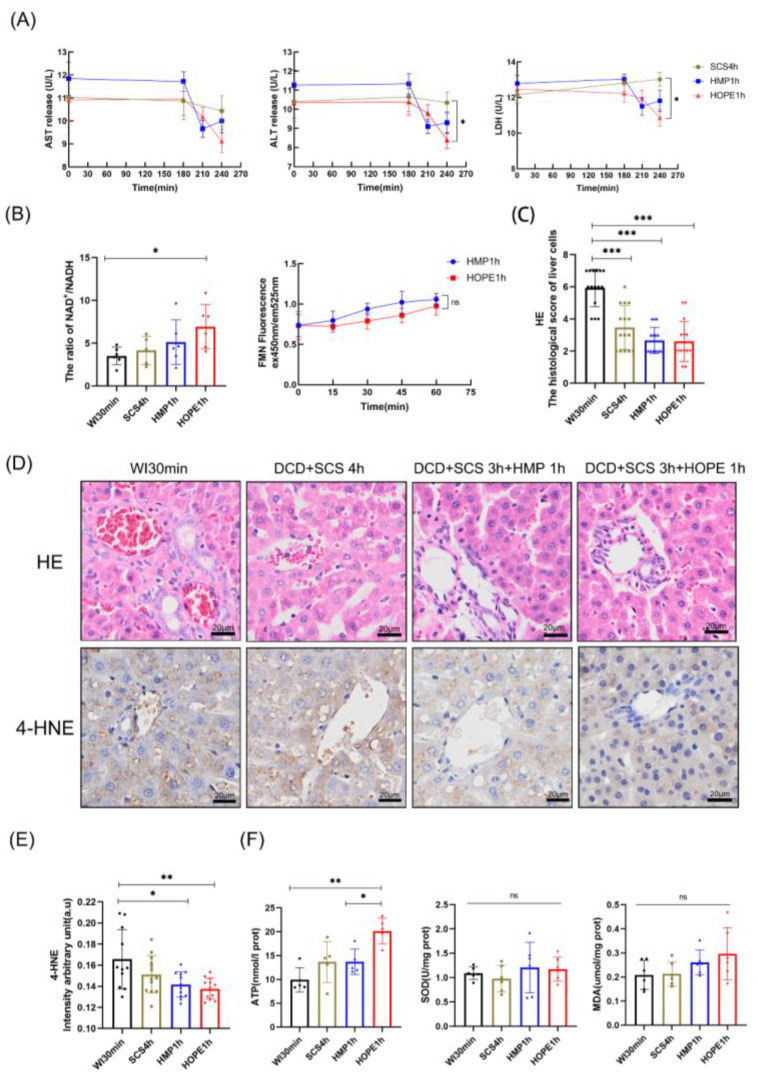
Liver function in the four groups in perfusion series 1. (**A**) ALT, AST, and LDH activity in the perfusate. Data were transformed to a log2 scale. (**B**) The ratio of NAD^+^/NADH in four groups. During hyperthymic perfusion for 1 h with fluorescence of FMN in perfusate every 15 min, detection of the emitted light was quantified at 525 nm, while excitation was performed at a wavelength of 450 nm. (**C**) Histological scores were analyzed based on Suzuki’s criteria. (**D**) H&E staining was performed on the paraffin-embedded sections of rat liver tissues. Scale bar: 20 μm; IHC staining of 4-HNE was performed on the paraffin-embedded section of rat liver tissues. Scale bar: 20 μm. (**E**) IHC stained 4-HNE were quantified using Image J. (**F**) ATP, SOD, and MDA were tested to evaluate mitochondrial function. (*n* = 6 per group for (**A**,**B**,**F**); for (**C**,**D**), *n* = 3 independent experiments). Mean ± SD, N.S., *p* > 0.05, * *p* < 0.05, ** *p* < 0.01, *** *p* < 0.001.

Figure 1A describes our experiment groups, Figure 1B shows the DCD process, and Figure 1D–F display the preservation and MP parameters. DCD liver subjected to 30 min warm ischemic following 4 h cold storage displayed a more mottled hemorrhagic pattern on the surface compared with DCD liver subjected to 3 h cold storage and subsequent MP, while HOPE-treated liver had a cleaner surface and better texture (Figure 1C). The release of liver-specific enzymes AST, ALT, and LDH was more specific to hepatocellular injury, and three markers in perfusate showed the decreased injury of DCD liver in three experimental groups during the storage and MP, while the HOPE 1 h group had lower levels of ALT (SCS 3506 ± 2368 vs. HMP 873 ± 618 vs. HOPE 402 ± 2023 U/L, respectively), and LDH (SCS 14,184 ± 56,212 vs. HMP 5353 ± 4684 vs. HOPE 2398 ± 1732 U/L, respectively) compared with the SCS 4 h and HMP 1 h groups (*p* < 0.05; Figure 2A). Microscopic tissue structure was assessed using hematoxylin and eosin (H&E) in a blinded manner. Liver in warm ischemic for 30 min without other interventions presented more several sinusoidal congestions, cytoplasmic vacuolization, and necrosis than the other three groups (*p* < 0.05; Figure 2C,D). The liver in the SCS 4 h group manifested moderate sinusoidal congestion and mild cytoplasmic vacuolization compared with the HMP 1 h and HOPE 1 h groups, while single-cell necrosis was seen in all groups; however, favorable morphological manifestations were observed for livers in the HMP 1 h and HOPE 1 h groups.

### 2.2. HOPE Enhanced Mitochondrial Function and Attenuated Oxidative Stress in DCD Liver

Complex I catalyzes the first step of NADH oxidations, elevating the NAD+/NADH ratio and translocating protons across the inner mitochondrial membrane, ultimately leading to energy production [29]. The HOPE 1 h group exhibited increased cellular NAD+/NADH ratios more than the three other groups, especially WI 30 min (Figure 2B). Flavin mononucleotide (FMN) is a marker of mitochondrial complex I injury that can be measured in real-time during perfusion [30,31]. FMN was assessed in perfusate every 15 min during hypothermic machine perfusion, and the results showed that FMN decreased more in the HOPE 1 h than the HMP 1 h group (Figure 2B). Mitochondrial function was determined by the ability to utilize oxygen and nutrients to generate ATP, and at the end of hypothermic storage/perfusion, liver samples in the HOPE group demonstrated higher levels than the other three groups (*p* < 0.05; Figure 2F). The antioxidant superoxide dismutase (SOD) and the lipid peroxidation metabolite malondialdehyde (MDA) increased in both the HMP and HOPE groups when compared with the other two groups (Figure 2F). As increased MDA was inversely correlated with liver function in machine perfusion groups, 4-hydroxynonenal (4-HNE), which is another marker of lipid peroxidation and a critical mediator of mitochondrial dysfunction, was significantly lower in the HOPE 1 h group after IHC staining (*p* < 0.05; Figure 2D,E).

### 2.3. HOPE Regulated Transcriptomic Profiles in DCD Liver

**Figure 3:**
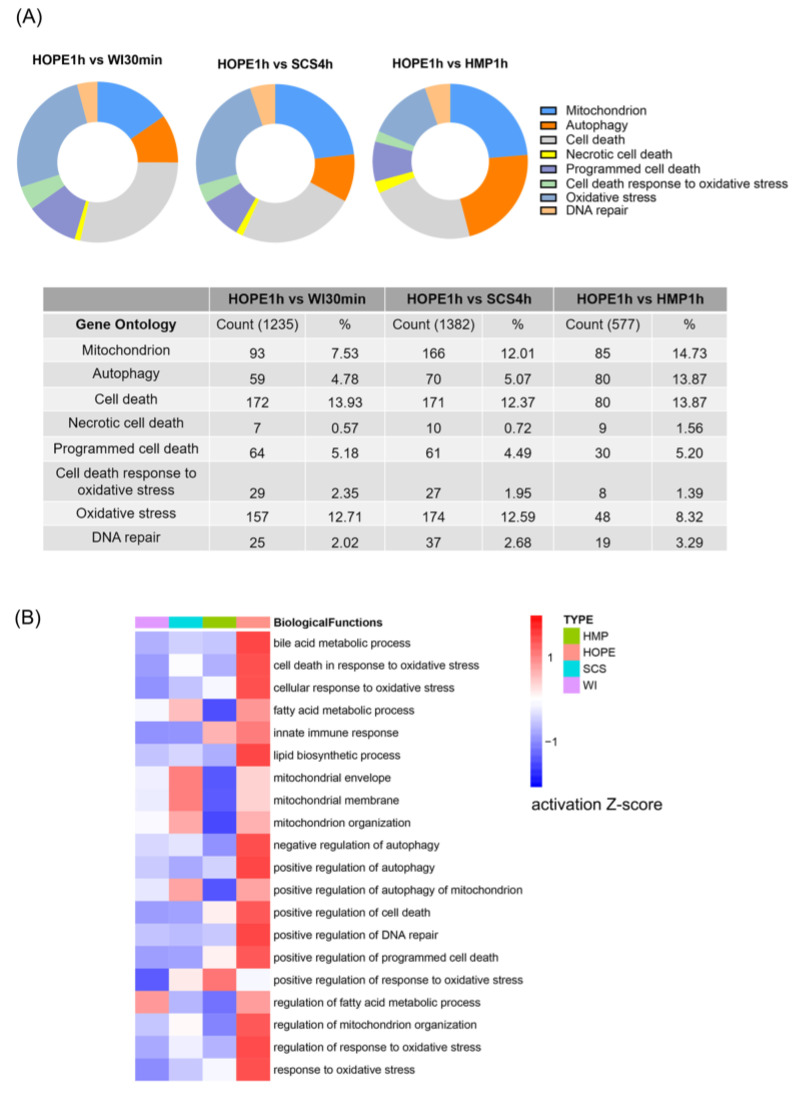
HOPE-regulated transcriptomic gene in DCD liver targeted mitochondrial bioenergetics, autophagy, and cell death. (**A**) The pie chart represents the most common gene ontology clusters that were altered in response to hypoxia-ischemic injury; tables represent the percentile change in gene ontology from the total genes in the array. (**B**) Heat map comparison analysis shows changes in gene expression related to a variety of biological functions across observations in multiple datasets. The 20 rows (biological function) with the highest total absolute z-scores across the set of observations were sorted. Four columns designate gene expression signatures in the four groups: WI 30 min, SCS 4 h, HMP 1 h, and HOPE 1 h. The activation z-scores for biological functions are displayed using a gradient from dark blue to red for activation z-scores. (*n* = 3).

To further test how HOPE may affect liver function following hypoxia-ischemic injury, we analyzed the liver transcriptome using RNA seq to clarify the vital quality control processes. Gene ontology mapping indicated that processes related to mitochondrial metabolism, oxidative stress, and cell death autophagy were increased in the WI 30 min, SCS 4 h and HMP 1 h groups compared with the HOPE 1 h group (Figure 3A). Consistent with these findings, the z-score was used to compare the four groups to observe changes in relevant downstream biological effects. The activation of biological functions displayed a gradient from dark blue to red for z-scores (Figure 3B). Pre-compiled lists of genes that are known to be involved in cellular biological functions were compared using different groups. HOPE resulted in the activation of bile metabolism and secretion, mitochondrial function, the autophagy pathway, cellular survival, and the regulation of oxidative stress.

### 2.4. HOPE Prevented Hepatocyte Injury and Reduced Apoptosis in Rat Liver

**Figure 4:**
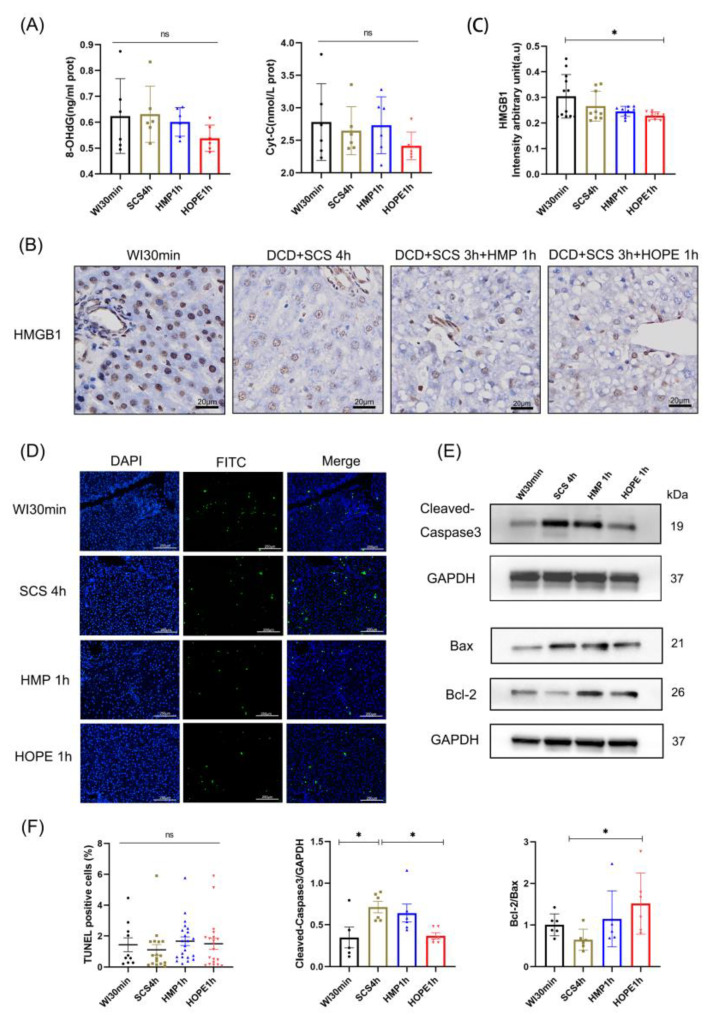
Nuclear damage and apoptosis induced by hypoxia-ischemic injury. (**A**) Enzyme-linked immunosorbent assay (ELISA) kit of 8-OHdG and Cyt C in liver tissue. (**B**) IHC staining of HMGB1 was performed on the paraffin-embedded section of rat liver tissues. Scale bar: 20 μm; (**C**) IHC-stained HMGB1 were quantified using Image J. (**D**) Apoptotic cells were evaluated using the TUNEL assay, and the number of apoptotic cells was counted. Scale bar: 250 μm; (**E**) Western blot shows the apoptosis-related protein level in four different groups; (**F**) TUNEL-positive cells and quantitative analysis of the relative intensity of Cleaved-Caspase 3, Bax, and Bcl-2 normalized to the GAPDH loading control were quantified using Image J (*n* = 6 per group for (**A**); *n* = 3 independent experiments for (**B**–**E**)). Mean ± SD, N.S., *p* > 0.05, * *p* < 0.05.

We detected several ischemic injuries in terms of nuclear injury, the release of 8-OHdG, and Cyt C lower in HOPE 1 h (Figure 4A). HOPE-treated liver grafts presented significantly less injury compared with the other three groups in terms of HMGB1 staining, especially WI 30 min (*p* < 0.05; Figure 4B,C). We used TUNEL staining for each liver tissue, and the ratios of positive cells to the total number of cells in the four groups showed no considerable differences (Figure 4D,F). Apoptosis-related proteins of cleaved-caspase3 and Bax were reduced in the HOPE 1 h group, the protein level of Bcl-2 was upregulated in the HOPE 1 h group, and the antiapoptotic of Bcl-2/Bax increased in the HOPE 1 h group more than the others, especially compared with SCS 4 h (*p* < 0.05; Figure 4E,F).

### 2.5. HOPE Restored Mitophagy and Promoted Mitophagy Flux after Hypoxia-Ischemic Injury in DCD Liver

**Figure 5:**
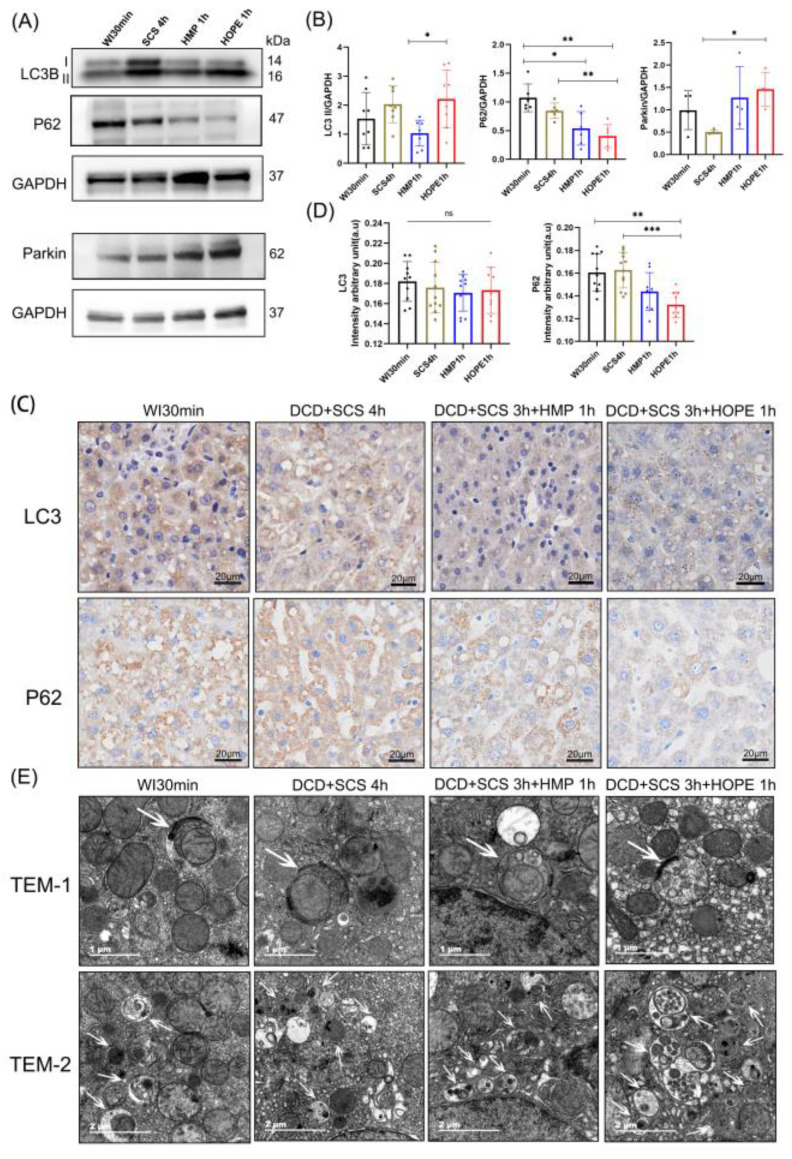
HOPE promoted mitophagy flux in DCD liver. (**A**) Western blot shows the mitophagy related protein levels; (**B**) the quantitative analysis of the relative intensity of LC3-II, P62, and Parkin normalized to the GAPDH loading control; (**C**) IHC staining of LC3 and P62 was performed on the paraffin-embedded section of rat liver tissues. Scale bar: 20 μm; (**D**) IHC-stained LC3 and P62 were quantified using Image J. (**E**) Electron micrographs of live tissue in four groups. White arrowheads in TEM-1: mitophagy vacuoles. White arrowheads in TEM-2: autophagy vacuoles. (*n* = 3 independent experiments for (**A**,**C**,**E**)). Mean ± SD, N.S., *p* > 0.05, * *p* < 0.05, ** *p* < 0.01, *** *p* < 0.001.

We investigated the association between hypoxic-ischemic liver and mitophagy. Hypoxia-ischemic injury induced autophagy, increased the expression of LC3-II/GAPDH in the four groups, and stimulated greater P62 accumulation in the WI 30 min and SCS 4 h groups compared with the HMP 1 h group, while the level dramatically decreased in the HOPE 1 h group (*p* < 0.05; Figure 5A,B), which means autophagy flux was blocked in WI 30 min and SCS 4 h and decreased in the HMP 1 h group. The immunohistochemistry of LC3 demonstrated hepatocyte LC3 staining in a pancytosolic pattern in the WI 30 min and SCS 4 h groups, whereas in the HMP 1 h and HOPE 1 h groups the LC3 staining changed to a granule-type staining pattern, indicating autophagosome formation, especially in the HOPE group (Figure 5C). The immunohistochemistry of P62 showed higher staining in a pancytosolic pattern in the WI 30 min and SCS 4 h groups, and P62 staining decreased in the HMP 1 h group, while it was lower in the HOPE 1 h group (*p* < 0.05; Figure 5C,D). The protein level and IHC staining results indicated that the HOPE 1 h group restored mitophagy and promoted mitophagy flux. Transmission electron microscopy (TEM) images of mitophagy vacuoles in TEM-1 or autophagy vacuoles in TEM-2 were observed in all four groups (Figure 5E, white arrow).

### 2.6. Mitophagy Was Essential for the Protective Effect of HOPE

**Figure 6:**
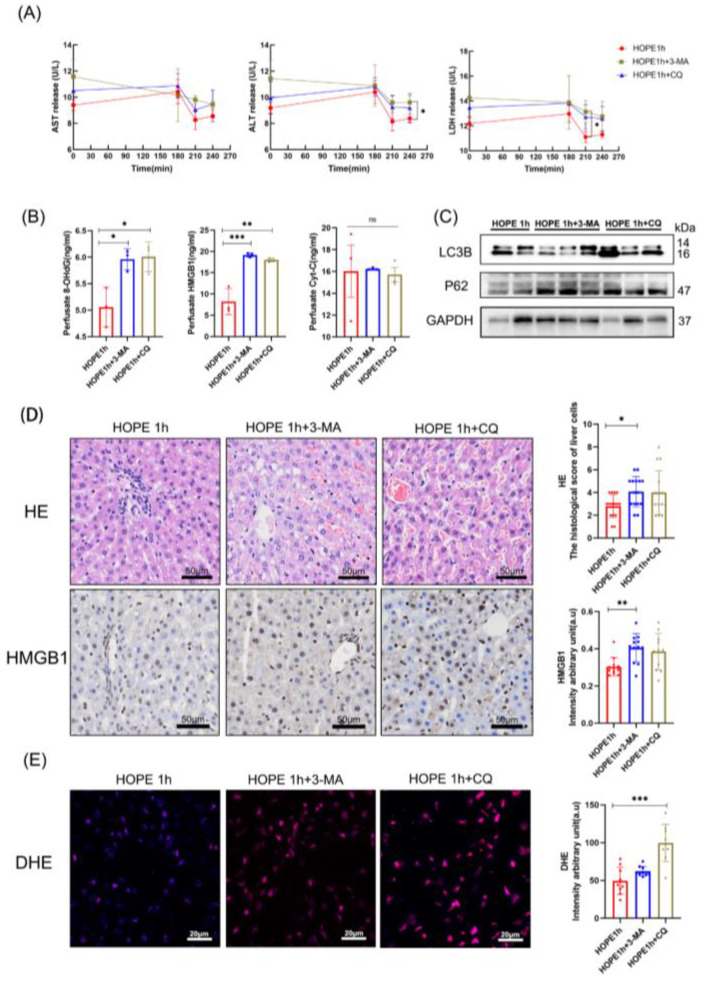
Autophagy inhibition attenuated the protective effect on DCD liver with HOPE 1 h. (**A**) The perfusate ALT, AST, and LDH data were transformed to a log2 scale. (**B**) 8-OHdG, HMGB1, and Cyt C in perfusate. (**C**) Western blot shows the autophagy-related protein levels. (**D**) H&E staining, scale bar: 50 μm; IHC staining of HMGB1, scale bar: 50 μm; histological scores were analyzed based on Suzuki’s criteria, and IHC-stained cells was quantified using Image J. (**E**) DHE staining was quantified using Image J, scale bar: 20 μm. (*n* = 3 independent experiments). Mean ± SD, N.S., *p* > 0.05, * *p* < 0.05, ** *p* < 0.01, *** *p* < 0.001.

To test whether mitophagy is required for the hepatocellular protective effect in the HOPE 1 h group to mitigate against hypoxic-ischemic injury, we used two autophagy inhibitors, 3-methyladenine (3-MA) and chloroquine (CQ), to block autophagy in both genotypes. The release of the liver-specific enzymes AST, ALT, and LDH in perfusate declined more slowly than in HOPE 1 h, especially for ALT and LDH (*p* < 0.05; Figure 6A). Autophagy inhibition presented with a significantly increased release of cell injury markers 8-OHdG, HMGB1, and Cyt C in perfusate (*p* < 0.05; Figure 6B), and 3-MA and CQ pretreatment significantly counteracted the protective effect of HOPE 1 h. The WB of autophagy levels in 3-MA and CQ groups represent the intervention success (Figure 6C). The microscopic tissue structure displayed increased sinusoidal congestions, cytoplasmic vacuolization, and necrosis in the autophagy-inhibition groups, and HMGB1 staining presented less injury in the HOPE 1 h group compared with the other two groups (*p* < 0.05; Figure 6D). Dihydroethidium (DHE) was often used for monitoring the formation of ROS in cells; the level of ROS was higher in autophagy-inhibition groups than in HOPE 1 h group. These observations suggest that HOPE 1 h protects the DCD liver in an autophagy-dependent manner.

### 2.7. HOPE Ameliorated Ischemic Reperfusion Injury in DCD Liver

**Figure 7:**
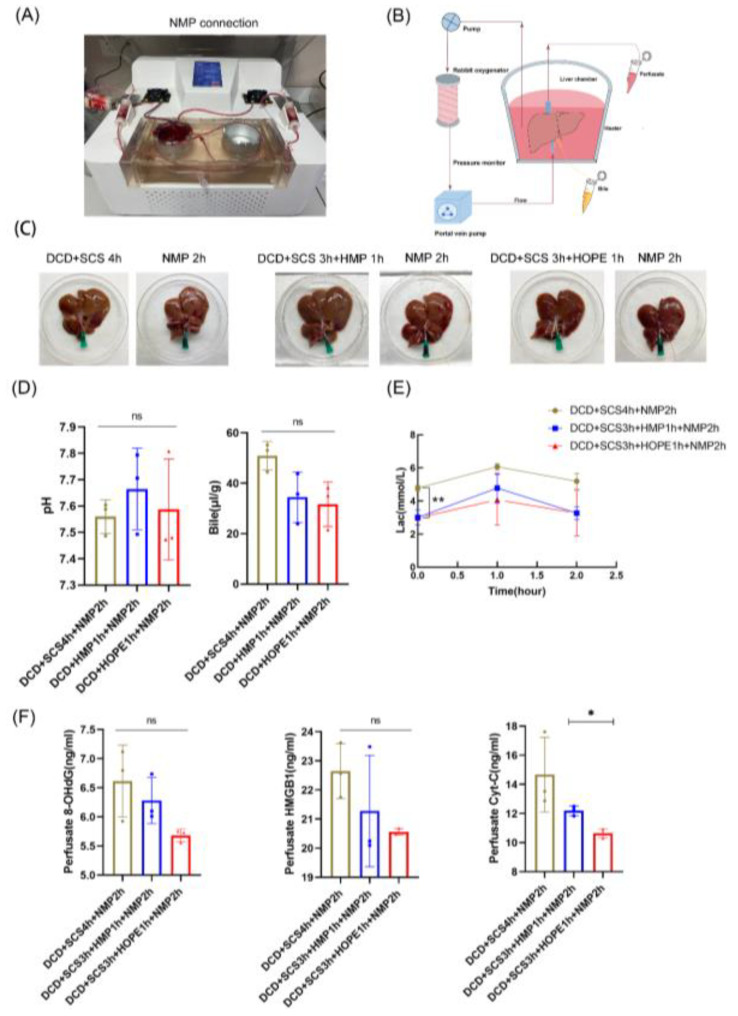
NMP following the hyperthymic storage/perfusion (*n* = 3). (**A**) NMP connection system. (**B**) Schematic representation of NMP devices. (**C**) liver appearance using three different hyperthymic storage or MP methods in perfusion series 2. (**D**) Bile pH and production. (**E**) Lactate concentration during NMP. (**F**) ELISA kit of 8-OHdG, Cyt C, and HMGB1 in perfusate (*n* = 3 independent experiments for **C**–**F**). Mean ± SD, N.S., *p* > 0.05, * *p* < 0.05, ** *p* < 0.01.

We applied NMP as a simulate transplantation, and all reached standard parameters, including the normothermic temperature and the presence of blood in the liver, and maintained homogeneous perfusion (Figure 7A,B; Figure S1A). By the end of NMP 2 h, there was no significant change in the texture and appearance of liver during simulated transplantation, although a small mottled hemorrhagic pattern appeared on the surface in some parts of the lobe (Figure 7C). Bile production decreased in all groups during simulated transplantation (Figure 7D), and was not significantly different in the SCS 4 h, HMP 1 h, and HOPE 1 h groups (50.9 ± 3.3 vs. 34.5 ± 5.8 vs. 31.6 ± 5.1 µL g^−1^, respectively), while the pH levels of bile in the three groups were all >7.5 (7.6 ± 0.04 vs. 7.7 ± 0.1 vs. 7.6 ± 0.1, respectively), and the results of bile acid metabolism were lower in the HOPE 1 h group (Figure S1B). Although the transaminase and LDH had no significant difference between the SCS 4 h, HMP 1 h, and HOPE 1 h groups (Figure S1C), the values at the end of NMP (ALT 335 ± 105 vs. 328 ± 121 vs. 257 ± 98 U/L, respectively; AST 502 ± 235 vs. 552 ± 136 vs. 386 ± 72 U/L, respectively; LDH 1696 ± 724 vs. 1264 ± 588 vs. 987 ± 143 U/L, respectively) were significantly degraded above hypothermic perfusion/storage. Liver-metabolized lactate levels in the SCS 4 h group were higher than in the HOPE 1 h group at the beginning of simulate transplantation; the levels all decreased in the three groups during NMP (*p* < 0.05; Figure 7E), and the average lactate levels at the end of NMP in the SCS 4 h, HMP 1 h, and HOPE 1 h groups were 5.2 ± 0.7, 3.3 ± 0.6, and 3.3 ± 1.9 mM, respectively. After NMP for 2 h, HOPE-treated livers presented with a significant decline in the release of 8-OHdG, HMGB1, and Cyt C, especially for Cyt C (*p* < 0.05; Figure 7F). HMGB1 staining presented less injury in the HOPE 1 h+NMP 2 h group compared with the other two groups (*p* < 0.05, Figure S1D).

## 3. Discussion

The application of MP before liver transplantation is considered a premier method to optimize liver availability and reduce ischemic reperfusion injury in high-risk grafts. In this study, we imitated the graft procurement and preservation protocol in clinical practice to investigate the potential protective mechanisms of HOPE in a DCD rat model compared with SCS and HMP. A short perfusion time of 1 or 2 h HOPE is sufficient to restore cellular ATP levels, recover mitochondrial function, and reduce cellular injury [32,33,34]. We hypothesized that mitophagy may play an important role in maintaining mitochondrial function in HOPE. Here, we discussed the potential HOPE mechanisms related to mitophagy that contributed to liver recovery.

Perfusate transaminases and mitochondrial complex I injury were correlated with subsequent graft performance. HOPE-treated DCD liver grafts were superior to those treated using SCS and HMP, with a high level of ATP and gradually declining hepatocellular injury (AST, ALT, and LDH). We demonstrated that the HOPE 1 h group displayed an enhanced cellular redox potential (NAD+/NADH ratio) by mitochondrial complex I activity, and FMN decreased in the HOPE 1 h group. Oxygen is the key aspect of hypothermic perfusion, and low-flow oxygen in HOPE does not provoke excess relevant ROS to release and prevent oxidative stress [17]. Excessive ROS can cause cell oxidative damage and mitochondrial dysfunction [35]. HOPE can reduce ROS production by increasing SOD and the decreasing 4-HNE staining; however, a high MDA value was observed after HOPE treatment. MDA is a stable lipid peroxidation metabolite, reflecting the free radical content and degree of lipid peroxidation [36]. However, active oxygenation downsides exist, especially during hypothermia, with intracellular redox-active iron ions triggering free-radical-mediated cell injury in the presence of oxygen [37]. Although there were no differences between the four groups, this suggests that ROS-mediated lipid injury during machine perfusion is indeed affected by oxygenation. A possible explanation might be postponed reoxygenation injury, but it had only minor effects that were not correlated to organ function after perfusion, and our results correlated with those of the study performed by Hoyer et al. [38].

We utilized transcriptomic analysis to investigate the cellular response to its microenvironment imposed by hypoxia-ischemic stress. Gene expression changed dramatically in different DCD graft interventions. At the transcriptome level, HOPE-treated DCD liver demonstrated more expression changes in genes responsible for bile metabolism, mitochondrial dynamics, cell survival, and the regulation of oxidative stress. HOPE is a simple preservation approach that is clearly protective against ischemic cholangiopathies [18], and the results of our z-score analysis regarding HOPE and bile metabolism and secretion pathways reflected its relationship with bile protection. A potential future investigation direction to study the mechanistic roles of proteins encoded by the genes identified in this study would be using experimental models with targeted molecules.

Many studies have provided evidence that hepatic autophagy is cytoprotective against IRI injury [24,39]. In our study, we found that hepatic protection during HOPE was associated with the activation of mitophagy via enhanced Parkin mitochondrial translocation and cell antiapoptotic. Parkin is an E3 ubiquitin-protein that is recruited from the cytoplasm to damaged mitochondria with low membrane potential via the accumulation of PTEN-induced kinase 1 (PINK1) and ubiquitinates-specific substrate proteins on the mitochondrial outer membrane [26]. P62 was the first autophagic cargo receptor identified in mammalian cells, and it plays a key role in mediating the formation and autophagic clearance of intracellular protein aggregates [40]. P62 can accumulate on the ubiquitinated mitochondrial matrix and then bind to LC3 to mediate the transport of ubiquitinated substrates into autophagosomes to initiate mitophagy [41]. The HOPE 1 h group showed enhanced mitophagy flux by increasing the LC3 II and Parkin protein levels while decreasing P62. LC3 staining clearly showed granule-type autophagosomes instead of pancytosolic patterns in the WI 30 min and SCS 4 h groups. TEM identified mitophagosomes, autophagosomes, and auto-lysosomes in the HOPE 1 h group. Conversely, in the WI 30 and SCS groups, autophagy flux was represented by upregulating both LC3 II and P62 protein levels, and decreasing Parkin protein levels in liver cells. We also established that elevated autophagy levels are required for the protective effect in the HOPE 1 h group because the inhibition of autophagy by 3-MA and CQ counteracted the hepatoprotective function of HOPE in DCD liver. Therefore, we demonstrated that HOPE can enhance mitophagy and promote mitophagy flux in DCD liver.

NMP was applied for simulating in vivo transplantation for 2 h, and we also found that a combination of end-ischemic ex situ HOPE and NMP resulted in a decreased release of transaminase and hepatocyte-related markers, indicating that the HOPE-NMP protocol might provide promising results for DCD liver treatment. The metabolized lactate was higher than 2.5 mM in our study, and the reason for this might be that we used whole blood for the complete 2 h instead of packed RBCs; however, the perfusate component still needs adjusting. HOPE-treated livers presented significantly less injury compared with cold storage and hyperthymic perfusion in terms of 8-OHdG, HMGB1, and Cyt C, which indicated that HOPE could alleviate ischemic reperfusion injury. In this study, the MP platform had some technical restrictions, so we could not compare end-ischemic D-HOPE applied for MP in DCD liver grafts, which is also a limitation of our study.

## 4. Materials and Methods

### 4.1. Ethical Statement

All research complied with ethical regulations, and the experimental protocol was approved by the Animal Experimental Ethical Inspection of the First Affiliated Hospital, Zhejiang University School of Medicine (Reference Number: 20221562). All authors had access to the study data and reviewed and approved the final manuscript.

### 4.2. Experimental Design

Sprague Dawley (SD) rats were anesthetized by cardiac arrest through incision of the diaphragm after being heparinized to simulate donor warm ischemia time for 30 min in situ. Liver graft procurement at the end of the abdominal aorta was flushed. The rats were randomly divided into four different groups to analyze the effect of different preservation strategies on graft injury and survival (*n* = 6 each).

(1) Warm ischemic group: livers were exposed to warm ischemia for 30 min without any other intervention (WI 30 min).
(2) Cold storage group: livers were exposed to warm ischemia for 30 min, followed by cold storage in Histidine-Tryptophan-Ketoglutarate (HTK) solution for 4 h (WI 30 min + SCS 4 h).
(3) HMP group: livers were exposed to warm ischemia for 30 min, followed by cold storage in HTK solution for 3 h, as well as 1 h of hypothermic machine perfusion (WI 30 min + SCS 3 h + HMP 1 h).
(4) HOPE group: livers were exposed to warm ischemia for 30 min, followed by cold storage in the HTK solution for 3 h and 1 h of hypothermic oxygenated machine perfusion (WI 30 min + SCS 3 h + HOPE 1 h).

3-MA/CQ: The rats were injected intraperitoneally with CQ (60 mg/kg) or 3-MA (15 mg/kg) (HY-17589A, HY-19312, MedChemExpress, Monmouth Junction, NJ, USA) at 1 h before warm ischemia. The same volume of blank solution was used as the HOPE 1 h control, and the livers were exposed to warm ischemia for 30 min, followed by cold storage in HTK solution for 3 h and 1 h of hypothermic oxygenated machine perfusion (HOPE 1 h + 3-MA, HOPE 1 h + CQ).

We performed NMP as a simulated transplantation following hypothermic storage/perfusion to assess the viability of the grafts (*n* = 3 each).

### 4.3. Liver Procurement

SD rats were socially housed in temperature- (18–23 °C) and humidity (50–70%)-controlled environments within filtered ventilated cages, with alternating 12 h light/dark cycles. Animals were provided with sterilized standard rat chow and water. Livers were procured from male rats (250–300 g, 6–8 weeks, Hangzhou, China). Rats were anesthetized; the liver was exposed using an abdominal median incision; the right renal vein, adrenal vein, gastric branches, and hepatic artery were all ligated; and rats’ bile ducts were cannulated with a catheter (0.45 mm, inner diameter); rats were heparinized (150 U) after the diaphragm was opened; and warm ischemia began at the time of cardiac arrest for 30 min in a warm environment. The liver was procured after flushing the abdominal aorta with 40 mL heparinized saline (50 U/mL), and the portal vein was cannulated with a 16-gauge catheter.

### 4.4. Cold Storage and MP Platform

The cold storage and MP platform allowed the grafts to be directly stored in a chamber with pressure, resistance, and temperature control. When operated with MP, the perfusate can be either recirculated or flushed with a single pass through the liver portal vein. Two oxygenators were used in this study, including the mouse oxygenator (Xijian Medical, Nanjing, China) for HOPE and the rabbit oxygenator (Xijian Medical, Nanjing, China) for NMP, and we used tubes of different thicknesses to adjust the portal flow (2–3 mm, inner diameter). A constant perfusion flow rate of HMP/HOPE was set at 1.2 mL/min, which correlated to our previous study [42]; additionally, the temperature was set to 4 °C, HOPE was oxygenated to a partial oxygen pressure (PO_2_) of 60–80 Kpa, and the total volume of HTK perfusate was 150 mL. The NMP technology provided continuous perfusion with oxygenated, packed full blood-based perfusate at 35–37 °C. According to Liu Yang et al. [43], the perfusate contained DMEM/F12, 20% FBS, 1% penicillin streptomycin solution (penicillin 10,000 U/mL, streptomycin 10,000 mg/mL), 5 U/mL of heparin, 2 U/L of insulin, and 2.5 mg/mL of dexamethasone. Full rat blood (3 or 4 rats, 30–45 mL) was reconstituted up to a total volume of 180 mL perfusate. Livers were perfused through the portal vein only, the flow rate was maintained at 2 mL/g/min using pressure control, and perfusates were actively oxygenated (PO_2_ 35 to 55 kPa). The system was primed using the same process, and HTK solution was flushed from the livers with 20 mL of cold saline before the livers were connected to the NMP.

### 4.5. Endpoints

#### 4.5.1. Perfusate Measurements

Perfusate was collected every 30 min during cold storage and hypothermic perfusion, and hourly during NMP. PO_2_, O_2_ saturation, pH, and lactate were measured in the outflow (intrahepatic vena cava) using an i-STAT blood analyzer (Abbott, Chicago, IL, USA). Serum aspartate transaminase/alanine transaminase (ALT/AST) and lactate dehydrogenase (LDH) concentrations were measured in the perfusate using (Automatic biochemical analyzer CS-1200, Guangzhou, China). FMN was also determined using a fluorescence assessment from perfusate samples which were centrifuged at 2000 rpm for 5 min to filter blood cells. Samples were analyzed using fluorescence spectroscopy (Varioskan Flash, Thermo, Waltham, MA, USA), and a monochrome light with an excitation wavelength of 450 nm and 525 nm emitted by the FMN was recorded with 100% gain. Cumulative bile production during NMP was collected using a 1.5 mL Eppendorf tube during the NMP.

#### 4.5.2. Liver Samples

The middle lobe of the left liver graft was taken at the end of the experiment, and specimens were either flash frozen in liquid nitrogen and stored at −80 °C or fixed in buffered 4% formaldehyde for at least 24 h. Flash-frozen tissues were used to quantify ATP, and the ATP assay kit was performed according to the manufacturer’s instructions (Beyotime, Shanghai, China). SOD is an important antioxidant that was measured using the Total Superoxide Dismutase Assay Kit with WST-8 (Beyotime, China), and MDA is a stable lipid peroxidation metabolite obtained using assay in the homogenates (Nanjing Jiancheng Bioengineering Institute, Nanjing, China). ATP, SOD, and MDA concentrations were normalized to tissue protein content determined using the BCA protein assay (Thermo Scientific, Waltham, MA, USA).

For H&E and immunohistochemistry, a small section of tissue from the middle lobe of the left liver was fixed in 4% formaldehyde, routinely embedded in paraffin, sectioned, stained with H&E, and then observed under a microscope. Cell-damage-related markers were HMGB1 (Abcam, ab79823, 1:350). Autophagy flux markers of LC3 (Proteintech, 14600-1-AP, 1:300), P62 (Abcam, ab155686, 1:500), and biomarkers for oxidative stress 4-hydroxynonenal (Abcam, ab46545, 1:300) were all measured using IHC staining. DAB was used as the chromogen, and the section was then counterstained with hematoxylin and mounted. Images were obtained using microscopy, and the percentage of the positive area was quantified using Image J software.

ELISA: In perfusate and tissue samples, 8-hydroxy-2′-deoxyguanosine (8-OHdG) is a marker of DNA oxidative damage; nuclear injury of high-mobility group box protein-1 (HMGB1) functions as an alarmin protein in response to inflammation; and Cytochrome C (Cyt C), which plays a critical role in cellular apoptosis and is also involved in the electron transport chain, can be detected using the ELISA Kit (Fankewei, Shenzhen, China).

DHE staining: The middle lobe of the left liver graft was taken at the end of the experiment, specimens were stored at −80 °C, frozen sections were used to quantify DHE, and the DHE assay kit was performed according to the manufacturer’s instructions (Beyotime, Nanjing, China).

Western blot: Standard Western blotting procedures were used. Antibodies used for Western blots included anti-LC3B (Cell Signaling Technology, 43566T, Danvers, MA, USA), anti-P62 (Abcam, ab155686, Cambridge, UK), anti-Parkin (Abcolonal, A11172, Woburn, MA, USA), anti-GAPDH (#5174, Cell Signaling Technology), anti-Cleaved Caspase-3 (#9664, Cell Signaling Technology), anti-Bax (Abmart, T40051, Berkeley Heights, NJ, USA), and anti-Bcl-2 (Abmart, T40056).

TUNEL: The TUNEL assay was performed on liver sections according to the description of the TUNEL Apoptosis Assay Kit (Beyotime, Nanjing, China). Samples were mounted in a media that contained DAPI. The total number of nuclei (blue) and TUNEL-positive nuclei (green) were counted under a fluorescence microscope (IX73, Olympus, Tokyo, Japan).

TEM: TEM was used to assess mitophagy, and liver tissues cut into 2–3 mm were fixed using 2% glutaraldehyde and 2% paraformaldehyde in 0.1 M PBS at 4 °C. Then, they were rinsed in PBS, post-fixed with 1% OsO4 and 0.1% K3Fe (CN)6, dehydrated through a graded series of ethanol and absolute acetone, and embedded in Spurr resin. Ultra-thin sections were cut using an ultramicrotome and stained using lead citrate. The structural mitophagy was assessed and then examined with a Hitachi Model H-7650 TEM.

RNA sequence and data analysis: Samples for RNA-sequencing (RNA-seq) were collected from liver tissue at baseline in four groups before stimulating transplantation. The total amount and integrity of RNA were assessed using the RNA Nano 6000 Assay Kit of the Bioanalyzer 2100 system (Agilent Technologies, Santa Clara, CA, USA). Total RNA was used as input material for the RNA sample preparations. To select preferential cDNA fragments of 370~420 bp in length, the library fragments were purified with the AMPure XP system (Beckman Coulter, Beverly, MA, USA). Then, through PCR amplification, the PCR product was purified using AMPure XP beads, and the library was finally obtained. The index of the reference genome was built using Hisat2 (v2.0.5), and paired-end clean reads were aligned to the reference genome using Hisat2 (v2.0.5). The mapped reads of each sample were assembled with StringTie (v1.3.3b) (Mihaela Pertea. et al. 2015) using a reference-based approach.

Exploratory analysis was conducted using various functions and packages from R and the Bioconductor project. Predicted downstream biological functions were compared among groups, and those with the highest total absolute z-scores across the set of observations were sorted.

### 4.6. Statistical Analysis

All graphics were produced and statistical analyses were performed with Prism 8.3.0 (GraphPad Software Inc., La Jolla, CA, USA). Data are presented as the mean ± SD of at least three independent assays. After testing for normality using the normality and lognormality test, group variables were analyzed with one-way analysis of variance or the Kruskal–Wallis test accordingly. Repeated measures of two-way ANOVAs were used for the comparison of the time-course perfusion data. *p* < 0.05 was considered statistically significant.

## 5. Conclusions

HOPE may trigger the activation of protective genes against early liver IRI, and our study offers a potential mechanism in which HOPE repairs mitochondria by effective mitophagy. Mitophagy may become a promising target for improving hypoxia-ischemic injury in DCD liver.

## Data Availability

The data that support the findings of this study are available from the corresponding author upon reasonable request. The transcriptomic profiles have been uploaded to the GEO database with No. GSE215134.

## Supplementary Materials

The following supporting information can be downloaded at: https://www.mdpi.com/article/10.3390/ijms24065403/s1.

## Abbreviations

HOPE, hypothermic oxygenated machine perfusion; DCD, donation after circulatory death; HMP, hypothermic machine perfusion; NMP, normothermic machine perfusion; MP, machine perfusion; SCS, static cold storage; IC, biliary ischemic cholangiopathy; PNF, primary nonfunction; D-HOPE, dual hypothermic oxygenated machine perfusion; ATP, adenosine triphosphate; ROS, reactive oxygen species; IRI, ischemic-reperfusion injury; WI, warm ischemic; HTK, histidine-Tryptophan-Ketoglutarate; AST, aspartate transaminase; ALT, alanine transaminase; LDH; lactate dehydrogenase; FMN, flavin mononucleotide; SOD, superoxide dismutase; MDA, malondialdehyde; 8-OHdG, 8-hydroxy-2′-deoxyguanosine; HMGB1, high mobility group box protein-1; Cyt C, Cytochrome C; 4-HNE, 4-hydroxynonenal; DHE, dihydroethidium; OXPHOS, mitochondrial oxidative phosphorylation; PINK1: PTEN-induced kinase 1.
