# Supplementary figures and images for: Hypothermic Oxygenated Machine Perfusion Promotes Mitophagy Flux against Hypoxia-Ischemic Injury in Rat DCD Liver
Source: Int J Mol Sci. 2023 Mar 11;24(6):5403. doi: 10.3390/ijms24065403 (PMC10049087; doi:10.3390/ijms24065403)

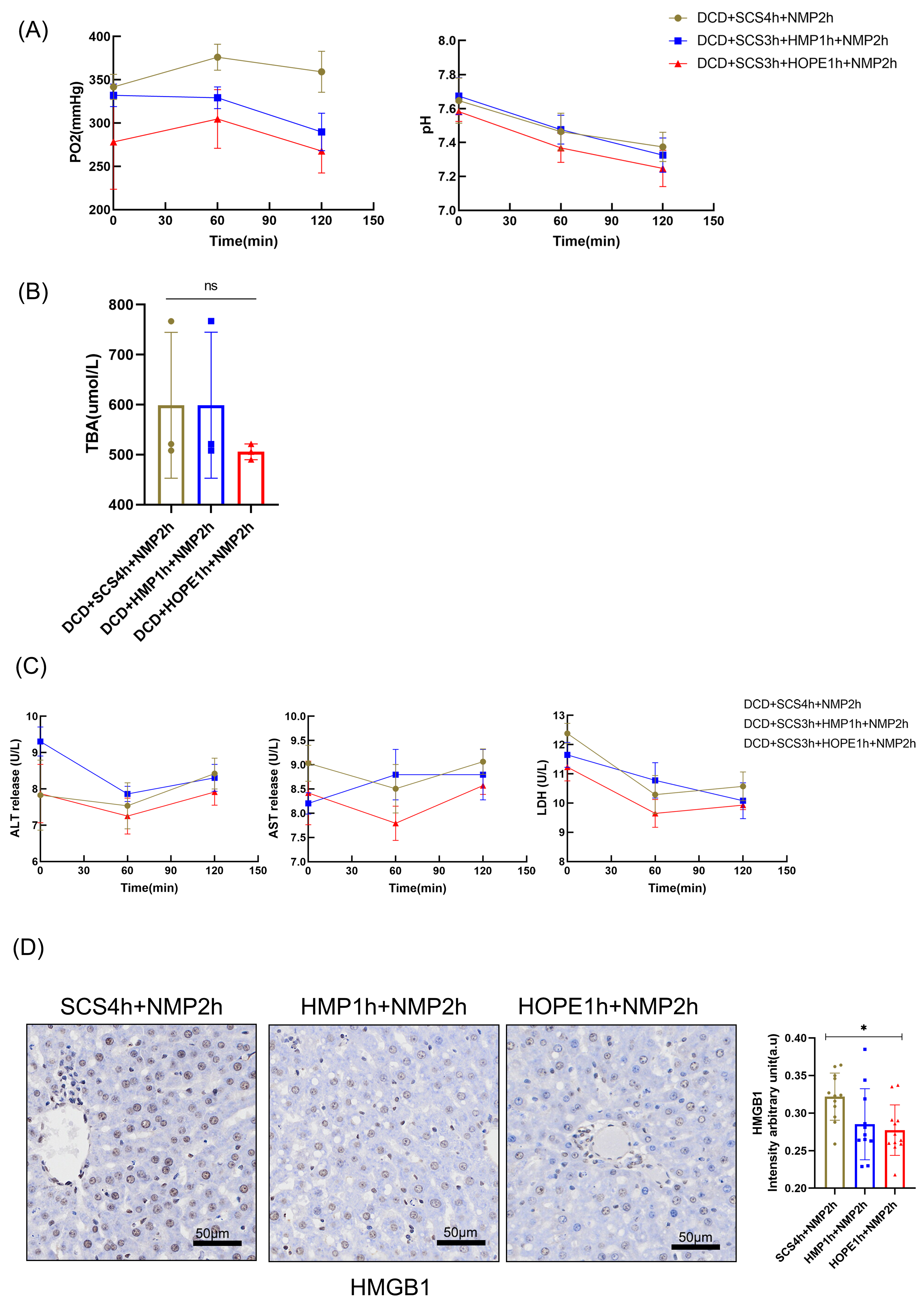

Supplement: Supplementary file 1 [file ijms-24-05403-s001.zip › Figure S1.tif]
